# A Novel Kunitz Trypsin Inhibitor from *Enterolobium gummiferum* Seeds Exhibits Antibiofilm Properties against Pathogenic Yeasts

**DOI:** 10.3390/molecules29163777

**Published:** 2024-08-09

**Authors:** Matheus M. da Silva, Caio F. R. de Oliveira, Claudiane V. Almeida, Ismaell A. S. Sobrinho, Maria L. R. Macedo

**Affiliations:** 1 Laboratório de Purificação de Proteínas e suas Funções Biológicas, Faculdade de Ciências Farmacêuticas, Alimentos e Nutrição, Universidade Federal de Mato Grosso do Sul, Campo Grande 79070-900, MS, Brazil; matheusmaganhadasilva@gmail.com (M.M.d.S.); clauvilharroel@gmail.com (C.V.A.); ismaellavelinos@gmail.com (I.A.S.S.); 2 Instituto Federal de Mato Grosso, Campus Guarantã do Norte, Guarantã do Norte 78520-000, MT, Brazil; oliveiracfr@gmail.com

**Keywords:** antibiofilm, characterisation, circular dichroism, peptidase inhibitor

## Abstract

Plant peptidase inhibitors play crucial roles in plant defence mechanisms and physiological processes. In this study, we isolated and characterised a Kunitz trypsin inhibitor from *Enterolobium gummiferum* seeds named EgPI (*E. gummiferum* peptidase inhibitor). The purification process involved two chromatography steps using size exclusion and hydrophobic resins, resulting in high purity and yield. EgPI appeared as a single band of ~20 kDa in SDS-PAGE. Under reducing conditions, the inhibitor exhibited two polypeptide chains, with 15 and 5 kDa. Functional characterisation revealed that EgPI displayed an inhibition stoichiometry of 1:1 against trypsin, with a dissociation constant of 8.4 × 10^−9^ mol·L^−1^. The amino-terminal sequencing of EgPI revealed the homology with Kunitz inhibitors. Circular dichroism analysis provided insights into the secondary structure of EgPI, which displayed the signature typical of Kunitz inhibitors. Stability studies demonstrated that EgPI maintained the secondary structure necessary to exhibit its inhibitory activity up to 70 °C and over a pH range from 2 to 8. Microbiological screening revealed that EgPI has antibiofilm properties against pathogenic yeasts at 1.125 μmol·L^−1^, and EgPI reduced *C. albicans* biofilm formation by 82.7%. The high affinity of EgPI for trypsin suggests potential applications in various fields. Furthermore, its antibiofilm properties recommended its usefulness in agriculture and antimicrobial therapy research, highlighting the practical implications of our research.

## 1. Introduction

Plant peptidase inhibitors (PIs) are proteins that play several roles in plant physiology. Some botanical families accumulate PIs as reserve proteins during the seed maturation process. PIs can modulate the endogenous enzymatic activities, protecting seed and embryo reserves against pathogens, including viruses, and bacteria [1,2,3,4]. An examination was conducted on peptidases and PIs in representative species from various plant groups. These molecules are targets of cancer, inflammation, and neurodegenerative diseases [4,5]. The PIs exhibited more significant variability than the peptidases themselves, demonstrating that PIs have adapted to regulate both internal and external peptidases [1].

PI classification follows the MEROPS database from Rawlings [5]. Peptidases and inhibitor species are grouped into different clusters in this database. Family and clan distribution encompasses 82 families and 39 clans, which exhibit sequence similarities and interrelate through tertiary structure [5]. Standard characteristics are common among plenty of PI families. Seeds from the Fabaceae family are a valuable source of PIs, mainly encompassing Kunitz and Bowman–Birk inhibitors. I3 Kunitz inhibitors are notably prevalent within the Fabaceae family, classified into three subfamilies: Mimosideae, Caesalpinioideae, and Papilionoideae [6].

Angiosperm species have the I3 Kunitz inhibitor family. These inhibitors are globular proteins with secondary structures consisting only of β-sheets, stabilised by various disulfide bonds [2,6]. Kunitz inhibitors exhibit a molecular weight of 18 to 24 kDa, with reports of smaller molecules (8 kDa) also being documented [4]. Kunitz inhibitors play a crucial role in inhibiting serine peptidases, such as trypsin and chymotrypsin, without affecting metallopeptidases [6]. The mechanism of action of these PIs is canonical, characterised by a rigid, reactive-site loop that binds to the enzyme’s active site, forming a stable and reversible complex [7]. A vital interaction occurs between Kunitz inhibitors and peptidases, especially with trypsin and chymotrypsin cases. This emphasises their potential for developing innovative biotechnological tools and therapeutic interventions. The inhibition of trypsin and chymotrypsin is essential in agricultural pest control, offering a sustainable pest management approach [8,9,10,11]. Kunitz inhibitors have shown significant inhibitory effects on pathogenic microorganisms, including yeasts. They specifically target and neutralise key proteolytic enzymes crucial for their growth and pathogenesis [12,13,14,15].

The genus *Enterolobium* belongs to the Mimosoideae family, where multiple Kunitz inhibitors have been isolated and characterised from seeds. These include the trypsin inhibitor from *Enterolobium contortisilliquum* (EcTI) [16,17] and the chymotrypsin inhibitor from *Enterolobium saman* [18]. Plant extracts from the *Enterolobium* genus are used for their anti-inflammatory and cytotoxic properties. Also, some species have been shown to have antibacterial, antifungal, and insecticidal activities. These activities primarily stem from the triterpenes or the phenolic compounds isolated from these plant extracts. Recently, PIs from the *Enterolobium* genus have displayed antimicrobial activities, such as *Enterolobium timbouva* trypsin inhibitor (EtTI) against *C. albicans*, *C. buinensis*, and *C. tropicalis* [15].

We are currently concentrating on the bioprospecting of additional trypsin inhibitors with potential therapeutic properties, focusing on species from the *Enterolobium* genus, which are prevalent in South and Central America. Among these species, *E. gummiferum* (Fabaceae, Mimosoideae) stands out as a widely distributed tree species in various Brazilian biomes, including the Amazon and Cerrado regions [19]. From *E. gummiferum* seeds, we isolated a new trypsin inhibitor named EgPI (*E. gummiferum* peptidase inhibitor). This study presents the purification, characterisation, and antibiofilm properties of EgPI, a member of the I3 Kunitz trypsin inhibitor family.

## 2. Results

### 2.1. Purification of E. gummiferum Peptidase Inhibitor

**Figure 1:**
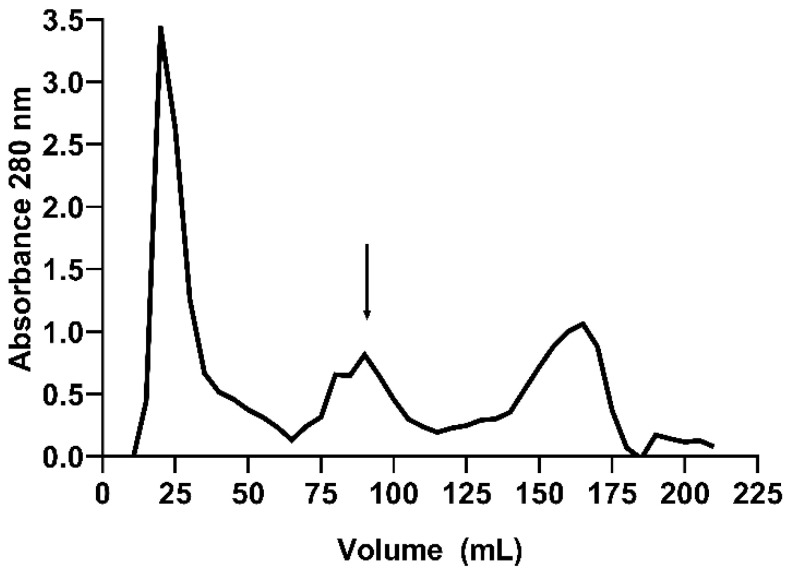
Gel filtration chromatography using a Sephadex G-100 column resulted in three peaks. The peak labelled EgPI G-100, which exhibited inhibitory activity against trypsin, is specified with an arrow.

The purification process of EgPI involved two chromatography steps. Inhibitory activity assays were conducted with bovine trypsin (Sigma-Aldrich, São Paulo, Brazil), showing increased specific activity from the crude extract (CE) to the peak from the Phenyl HP column (Cytiva, São Paulo, Brazil) (Table 1). Size exclusion chromatography using a Sephadex-G100 column (Cytiva, São Paulo, Brazil) separated the CE into three peaks (Figure 1). Inhibitory activity assays revealed that peak 2 (EgPI-G100) exhibited the highest inhibitory activity against trypsin. However, 12.5% SDS-PAGE analysis suggests that this peak contained two major proteins with similar molecular weights (~16 and ~20 kDa). As a polishing step, a hydrophobic chromatography column was used to purify EgPI.

**Figure 2:**
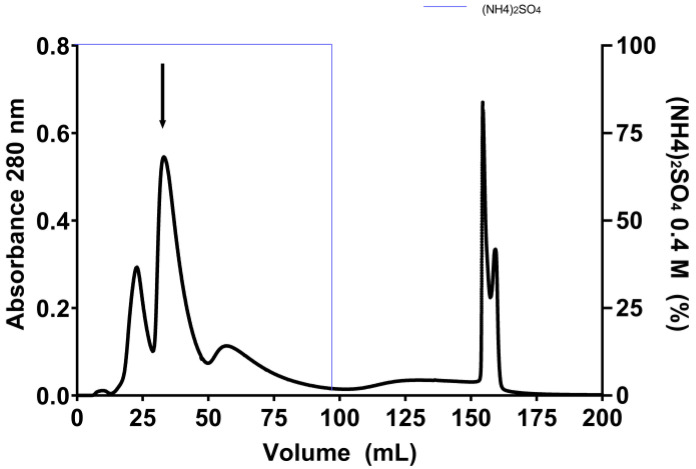
Hydrophobic chromatography using a Phenyl HP column results in five peaks. The gradient of (NH_4_)_2_SO_4_ is depicted with a blue line. An arrow marks the identification of the peak of EgPI, showing signs of inhibiting trypsin.

In the subsequent polishing phase, the Phenyl HP column efficiently resolved the EgPI-G100 peak from the Sephadex-G100 column, yielding five well-defined peaks (refer to Figure 2). The elution of the first three peaks was accomplished using a 0.1 M sodium phosphate buffer containing 0.4 M (NH_4_)_2_SO_4_ at pH 7.6. The washing process entailed using distilled water to extract the two remaining adsorbed peaks. Using salt in and salt out effects through the (NH_4_)_2_SO_4_ modulation facilitated the separation into five peaks. Based on inhibitory activity assays against trypsin, only peak 2 demonstrated inhibitory activity and was identified as EgPI.

**Figure 3:**
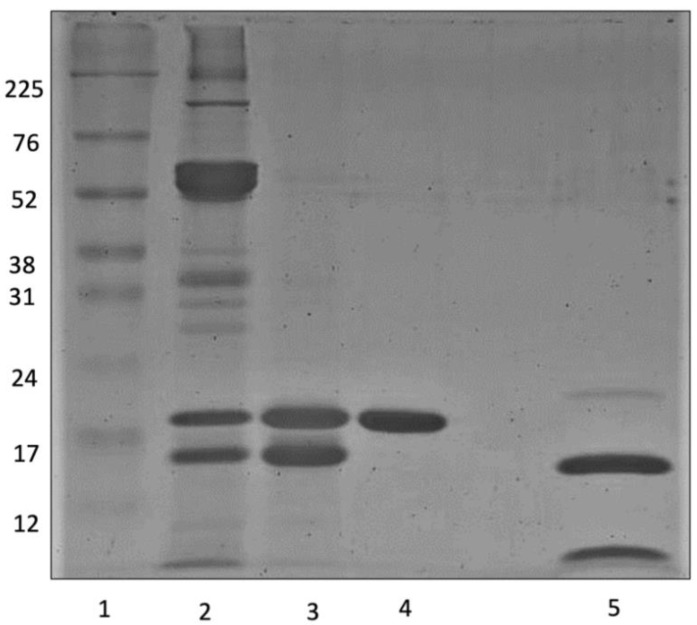
The 12.5% SDS-PAGE shows the results of the purification steps: 1: high-range molar mass marker (kDa); 2: crude extract; 3: EgPI-G100; 4: EgPI; and 5: EgPI reduced with 0.1 mol·L^−1^ DTT.

The EgPI purification process was monitored using SDS-PAGE (Figure 3). Hydrophobicity chromatography achieved a high-purity molecule, as EgPI displayed a single band at ~20 kDa under non-reducing conditions. Upon reduction with DTT, the sample profile revealed two bands corresponding to polypeptide chains of approximately 15 and 5 kDa. The purification table (Table 1) states an increase in specific activity and fold, yielding 56%.

### 2.2. Inhibitory Properties of EgPI

**Figure 4:**
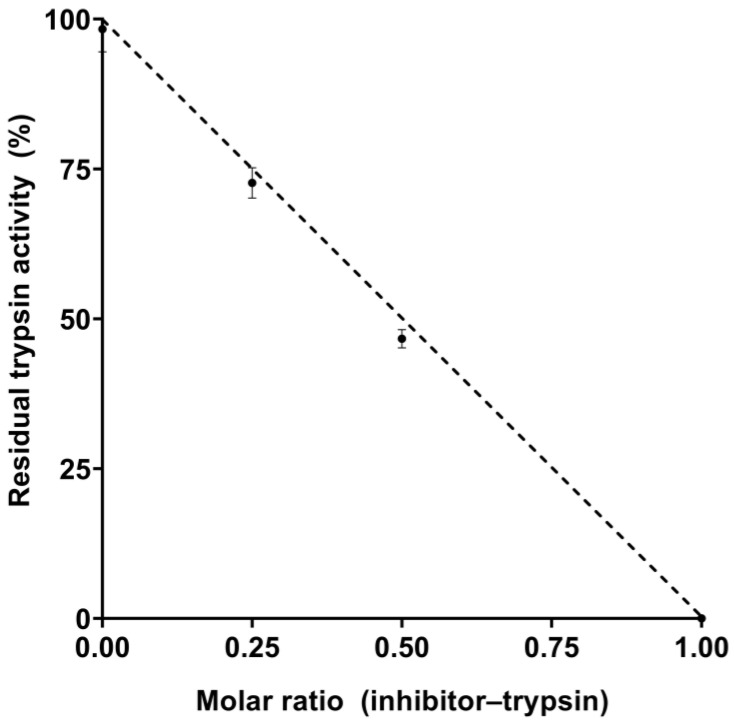
Stoichiometry of the inhibition of bovine trypsin by EgPI, demonstrating the relationship between inhibitor and enzyme molar ratios.

EgPI showed potent activity against bovine trypsin but exhibited only mild inhibition against chymotrypsin. Based on these results, the inhibitory characterisation properties focused on trypsin. We conducted enzymatic assays to establish the inhibition stoichiometry and the dissociation constant (Ki) of EgPI about trypsin. A consistent inhibition pattern was observed in experiments where different ratios of EgPI to trypsin were used. Complete inhibition was achieved when the ratio of the inhibitor to the enzyme was 1:1, a characteristic of PIs with a single reactive site (see Figure 4). Upon further analysis, it was found that the Ki value of EgPI and trypsin is 8.4 *×* 10^−9^ mol·L^−1^, which shows a strong affinity between them. This is a characteristic of competitive Kunitz inhibitors.

### 2.3. Amino-Terminal Sequencing

Amino-terminal sequencing determined the first 25 amino acids of EgPI. The alignment conducted with NCBI-BLAST (National Center for Biotechnology Information, Bethesda, MD, USA) confirmed that EgPI is part of the Kunitz family, showing the highest sequence identity with the inhibitor from *Enterolobium countorsiliquum*—EcTI [20]. Also, the alignment showed similarity between EgPI and Kunitz inhibitors found in *Acacia confusa* (AcTI) [21] and *Prosopis juliflora* (PjTI) [22] (Table 2). Identical amino acids were found at positions 4, 5, 8, 11, 14, 15, 17, 18, and 20, with conserved substitutions highlighted in dark grey, as shown in Table 2.

### 2.4. Analysis of the Secondary Structure and Stability of EgPI Using Circular Dichroism

**Figure 5:**
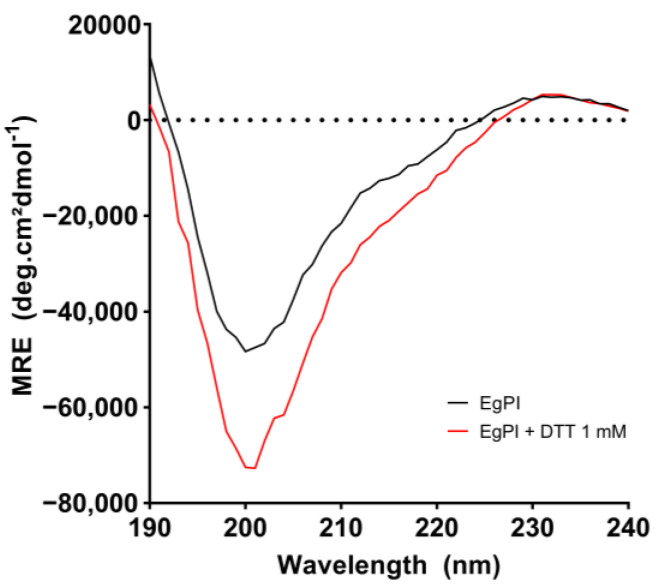
CD spectra of EgPI (black) were incubated with 1 mmol·L^−1^ DTT for 30 min at 30 °C.

The analysis of EgPI using circular dichroism (CD) spectroscopy revealed a characteristic far-UV spectrum with a minimum signal at approximately 200 nm and a maximum signal around 230 nm (Figure 5). The CONTIN algorithm was used to determine the secondary structure content, as summarised in Table 3. Under native conditions, EgPI exhibited a combination of β-sheet and random coil structures. However, incubation with DTT significantly reduced the β-sheet content from 21.6% to 8.3%. Therefore, the percentage of β-sheets was used as an indicator to assess the stability of EgPI under different temperature and pH conditions.

**Figure 6:**
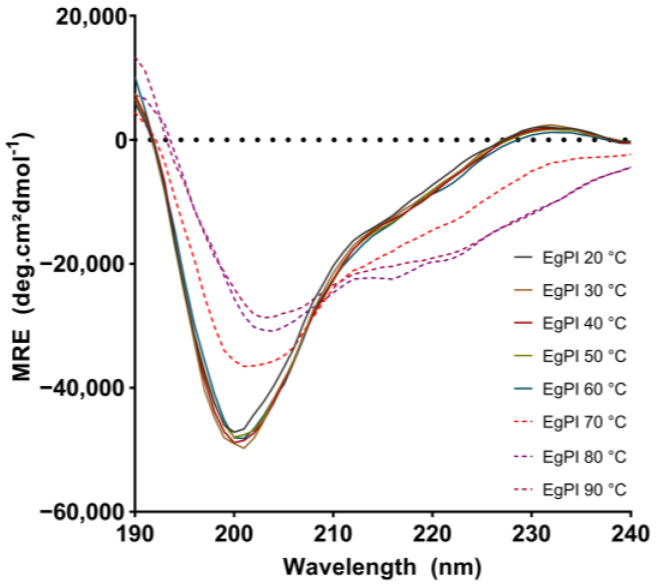
CD spectra of EgPI at various temperatures: 20 °C (grey); 30 °C (yellow); 40 °C (dark red); 50 °C (green); 60 °C (blue); 70 °C (red); 80 °C (purple); and 90 °C (pink). A notable reduction in β-sheet content and increased α-helix characteristics are observed starting at 70 °C (dashed lines).

The CD spectrum of EgPI remained consistent across temperatures ranging from 20 °C to 60 °C (Figure 6). At 70 °C, incubation caused notable changes in the CD spectrum. This included a significant reduction in the signal at 200 nm and the emergence of a negative signal at 230 nm. The decrease in the β-sheet content at 70 °C was equivalent to that observed when EgPI was incubated with DTT (Table 3). This suggests that inhibitory activity was nullified at this temperature. The reduction in β-sheet content has been associated with the loss of inhibitory activity in Kunitz inhibitors, as seen in examples such as Potato Trypsin Inhibitor and EATI [23,24]. When the temperature increased, the denaturation process caused an increase in the α-helix content (Table 3), as shown by a further decrease in signal at 200 nm and alterations in the spectra between 210 and 230 nm (Figure 6).

**Figure 7:**
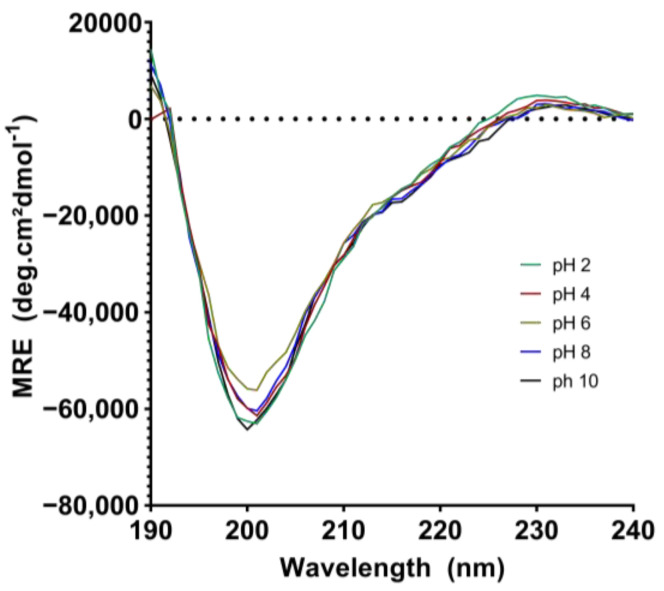
CD spectra of EgPI at various pH levels: pH 2 (green), pH 4 (red), pH 6 (yellow), pH 8 (blue), and pH 10 (black).

The CD spectral analysis revealed that EgPI’s secondary structure remained intact within a broad pH range (2 to 8) at the different pH levels (Figure 7). Despite this, there was a slight reduction in β-sheet content at pH 10, resembling the decreases observed during DTT reduction or heat denaturation. Across a wide range of pH values, this study found that EgPI exhibited remarkable stability (Table 3). Notably, inhibitory efficiency was significantly impacted when the β-sheet content fell below 10%. Comparable changes in CD profiles and concurrent activity loss have been documented for other PIs, such as TcTI and EATI, under reducing conditions and high temperatures [23,25].

### 2.5. Antifungal Properties

The antimicrobial properties of EgPI were assayed up to a concentration of 25 μmol·L^−1^. No antimicrobial activity was observed at this concentration. Further assays were conducted to determine the minimum biofilm inhibitory concentration (MBIC), and the minimum biofilm eradication concentration (MBEC). The result revealed that EgPI exhibited MBIC against *C. albicans* ATCC 5314 and *C. tropicalis* ATCC 750 biofilm at 11 µmol·L^−1^ and 5.5 µmol·L^−1^, respectively (Table 4).

**Figure 8:**
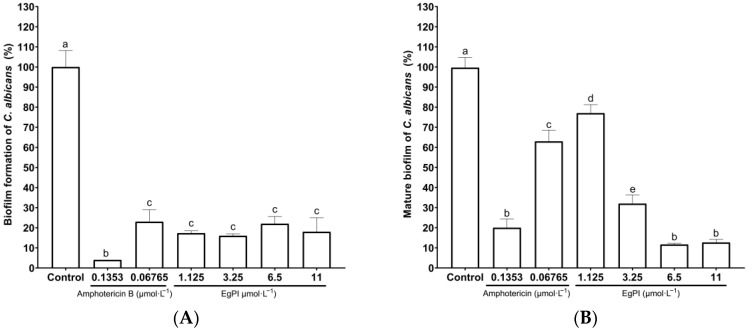
Effects of EgPI on the biofilm mass of *C. albicans* ATCC 5314. This experiment examined how EgPI and amphotericin B affected the formation (**A**) and mature biofilm (**B**). The data show the viable mass of biofilm attached to a flat-bottom well stained with crystal violet. The letters denote significant differences between the groups. These differences were determined using one-way ANOVA followed by post hoc analysis (*p* ≤ 0.05).

Serial dilutions were performed to analyse the impact of EgPI on the biofilm of *C. albicans* and *C. tropicalis*. The standard drug amphotericin B showed a dose-dependent antibiofilm effect on *C. albicans* (Figure 8A), whereas EgPI displayed an inhibitory effect at a concentration of 1.125 µmol·L^−1^. The impact of EgPI on mature *C. albicans* biofilm was also dose-dependent (Figure 8B). Notably, the reduction in biofilm mass reached 90% at the highest concentration tested.

**Figure 9:**
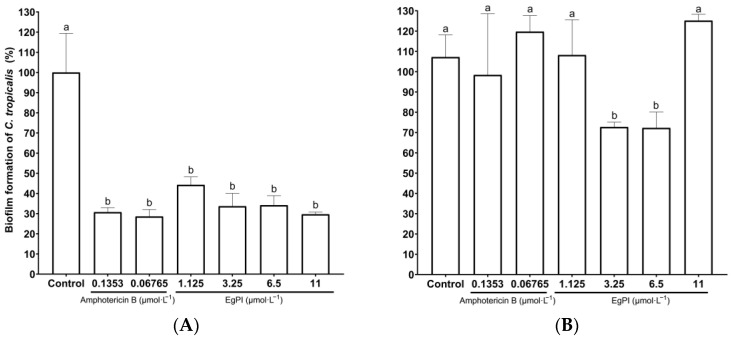
An investigation into the effects of EgPI and amphotericin B on the formation (**A**) and mature biofilm (**B**) of *C. tropicalis* ATCC 750 was conducted. The results present the viable biofilm adhered to a flat-bottom well stained with crystal violet. The letters denote significant differences between groups, determined using one-way ANOVA followed by post hoc analysis (*p* ≤ 0.05).

Regarding *C. tropicalis* biofilm, EgPI exhibited a similar inhibitory pattern to amphotericin B, reducing biofilm mass by approximately 70% (Figure 9A). The effect on mature *C. tropicalis* biofilm was observed at 5.5 and 2.75 µmol·L^−1^ (Figure 9B), resulting in a reduction of approximately 30% in biofilm mass. Interestingly, amphotericin B did not affect mature *C. tropicalis* biofilm.

**Figure 10:**
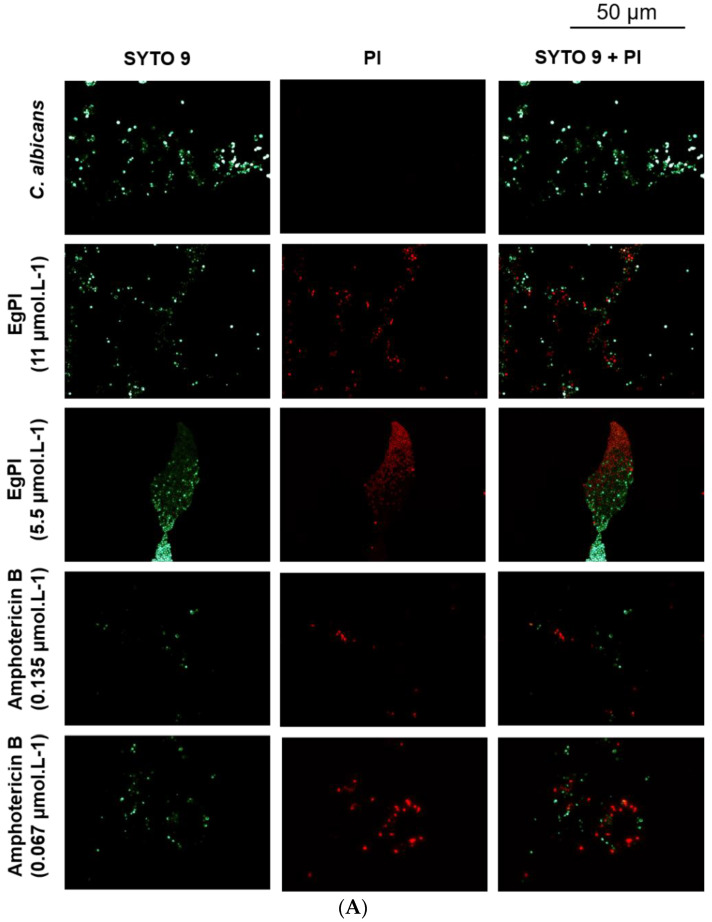

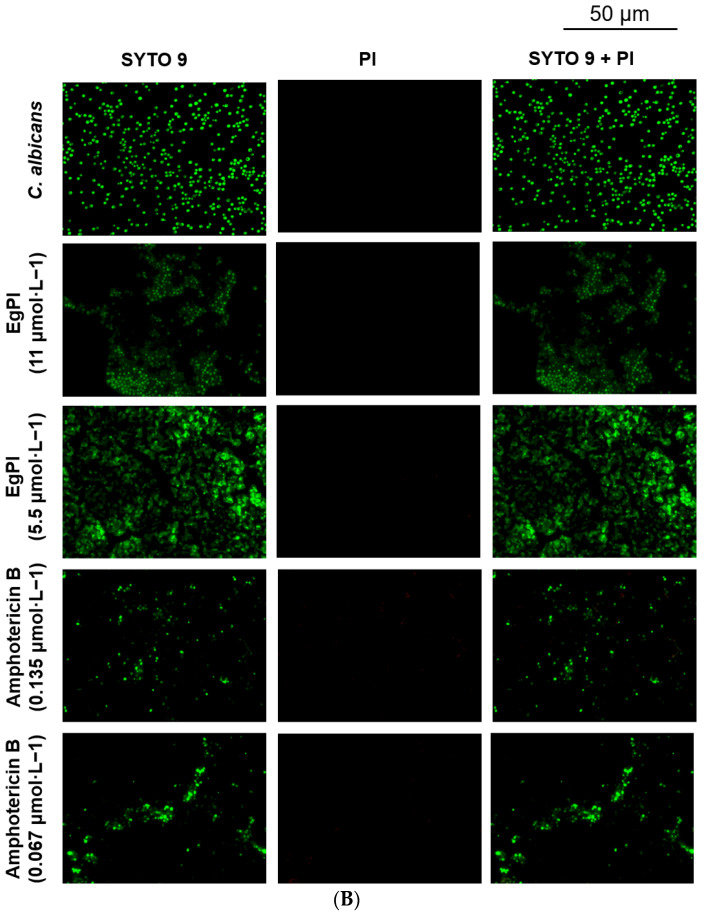
Fluorescence microscopy of the effect of EgPI on the formation (**A**) and mature (**B**) *C. albicans* biofilm. Viable cells are stained green, and non-viable cells are stained red.

Since the crystal violet assay corroborated the antibiofilm properties exhibited by EgPI, we further investigated the inhibitor’s effect on biofilm mass architecture and viability using fluorescence microscopy (Leica DM2000 LED) equipped with a Leica DFC7000 T camera and LAS V4.12 software (Leica Microsystems, São Paulo, Brazil). Both EgPI and amphotericin B prompted the reduction in viable cells and biofilm mass during the formation of *C. albicans* biofilm (Figure 10A). However, the analysis of mature *C. albicans* biofilm revealed the absence of non-viable cells, suggesting a disruptive effect without affecting cell viability (Figure 10B).

**Figure 11:**
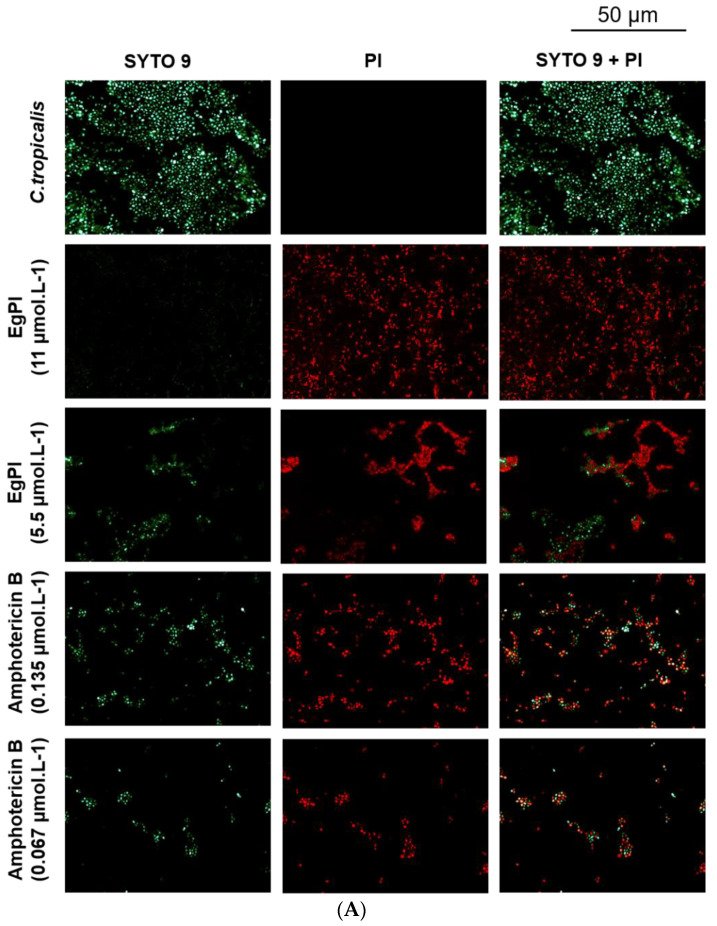

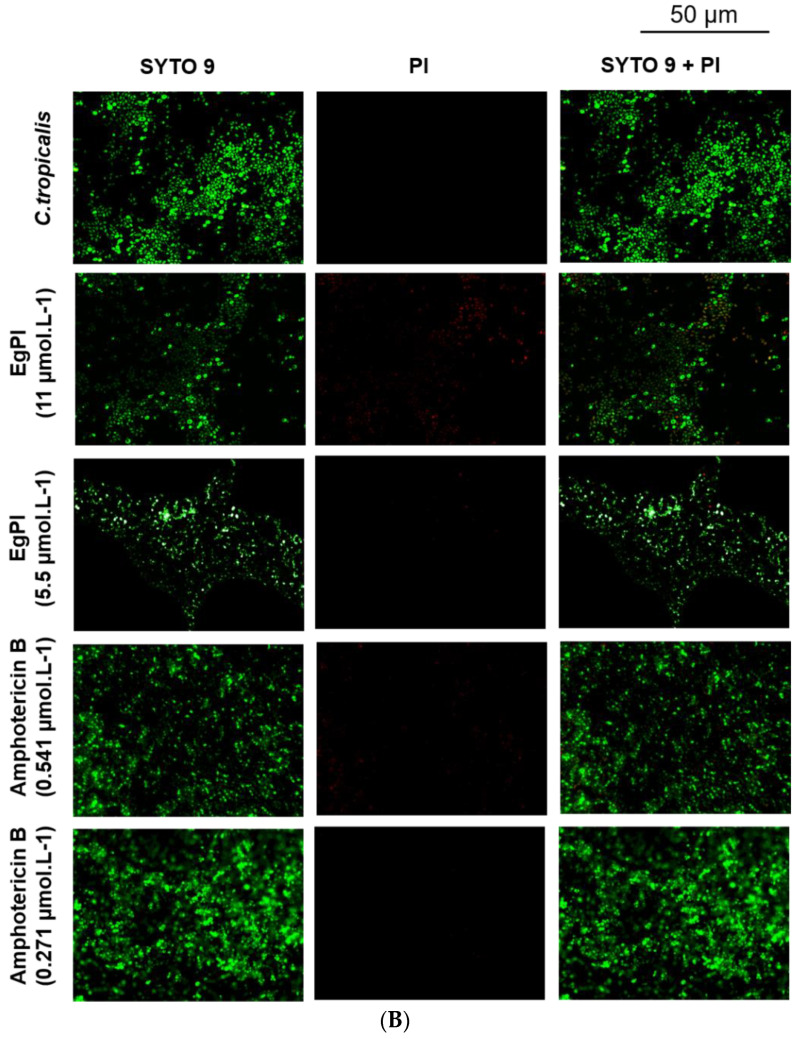
Fluorescence microscopy of the effect of EgPI on the formation (**A**) and mature (**B**) *C. tropicalis* biofilm. Viable cells are stained green, and non-viable cells are stained red.

The effect on *C. tropicalis* biofilm followed the pattern observed in *C. albicans* (Figure 11), with the presence of non-viable cells only during biofilm formation (Figure 11A). When the assay was performed with mature biofilm, no detection of red-stained cells was noticed (Figure 11B).

## 3. Discussion

Seeds of the Fabaceae family are a rich source of PIs, primarily comprising Kunitz and Bowman–Birk inhibitors. According to the MEROPS Database, the Kunitz inhibitor (I3) subfamily is abundant in Fabaceae, and includes three subfamilies: Faboideae, Caesalpinoideae, and Mimosoideae [6]. The genus *Enterolobium*, which belongs to the Mimosoideae subfamily [26], is notable for its I3 inhibitors. These inhibitors exhibit a wide range of activities against various peptidases involved in digestive processes, such as trypsin, blood coagulation factors, and inflammation. Most Kunitz inhibitors have a molar mass of 10–25 kDa, and exhibit diverse patterns of disulfide bridges and polypeptide chains [12]. Typically, the I3 subfamily of Kunitz inhibitors possesses four cysteine residues that form two disulfide bridges, with inhibitors categorised as having either a single or double polypeptide chain [6].

### 3.1. Inhibitory Activity and Ki Determination of EgPI

The purification of EgPI from crude extract using gel filtration chromatography was crucial for removing excess pigmentation from the sample. Pigmentation and high-molecular-weight proteins were eluted in the first peak, as observed in the SDS-PAGE. Gel filtration effectively concentrated EgPI in peak 2. However, SDS-PAGE expressed the existence of impurities with a molecular weight comparable to EgPI.

Hydrophobic chromatography was used as a polishing step to capitalise on the variations in surface hydrophobicity among proteins for separation. This method preserved the biological activity of EgPI using conditions and matrices that minimally denatured the proteins. The salting-out effect of ammonium sulphate played a crucial role in this process. By using a moderate concentration of ammonium sulphate, three peaks with different elution volumes were successfully separated [27].

We utilised SDS-PAGE and inhibitory activity assays to confirm that the second peak contained EgPI in its pure form. The purification process resulted in EgPI being produced with a remarkably high yield. SDS-PAGE analysis revealed that, under non-reducing conditions, EgPI had a relative molar mass of approximately 20 kDa. However, under reducing conditions, it exhibited two polypeptide chains. The molar mass pattern and the two polypeptide chains align with those observed in other I3 subfamilies of Kunitz inhibitors, particularly within the Mimosoideae subfamilies [6,20,28]. EgPI-specific activity towards trypsin was 13.17 × 10^4^ TIU·mg^−1^, yielding 56%.

The purification protocol and the quantity achieved are critical for proteins that exhibit biological functionality. Accurately measuring purity and yield values is crucial to guarantee the reproducibility of biological assays, especially when dealing with proteins that have biological activity. These proteins must be present in large amounts before they can be deemed suitable for biotechnological purposes.

### 3.2. Inhibitory Activity and Ki Determination of EgPI

Inhibitory activity against trypsin and chymotrypsin was assessed at each purification step. A consistently high inhibitory activity against trypsin was observed throughout all stages, while only minimal inhibitory activity against chymotrypsin was detected. Some Kunitz inhibitors are specific to a single enzyme [20], while others can inhibit multiple serine peptidases [29]. Some inhibitors, like ApTI and PdKI-2, can even target two classes of peptidases, inhibiting trypsin and papain [29,30]. It is plausible that a reactive site on EgPI may exhibit a weak interaction with chymotrypsin.

Stoichiometry studies of EgPI against trypsin showed a linear inhibition, with 100% inhibition in a 1:1 molar ratio of enzyme to inhibitor, a characteristic of single-headed Kunitz inhibitors. Further analysis, including Michaelis–Menten, Dixon, and Lineweaver–Burk, revealed a K*i* value of 8.40 × 10^−9^ mol·L^−1^. These data are consistent with other Kunitz inhibitors, such as EATI (1.75 × 10^−9^ mol·L^−1^) [23], TcTI (4.08 × 10^−9^ mol·L^−1^) [25], EcTI (0.88 × 10^−9^ mol·L^−1^) [31], and STI (3.20 × 10^−9^ mol·L^−1^) [32].

### 3.3. Amino-Terminal Identity

Kunitz inhibitors (I3) exhibit a highly conserved primary structure [16]. This conservation is notably similar to other I3 inhibitors, such as EcTI, which are characterised by four cysteine residues and two polypeptide chains [20]. The structural features are also found in AcTI [21] and PjTI [22]. Further emphasising this conservation, the amino-terminal region of the initial 25 amino acids of EgPI has a substantial similarity with Kunitz inhibitors, specifically showing 68% similarity with EcTI, 64% with AcTI, and 60% with PjTI.

When EcTI, STI, and Tamarind Kunitz Inhibitor (TKI) crystalised structure was superimposed, the β-sheet portions overlapped with a low RMSD value. However, the loop regions displayed a high RMSD value, suggesting that these portions are more variable among the Kunitz inhibitors. The reactive site loop is an exception to this variability, maintaining a similar shape across different Kunitz inhibitors [31]. Given the structural similarity among the I3 subfamily of Kunitz inhibitors, our CD analysis exhibits that EgPI shares a similar secondary structure, particularly in the β-sheet regions.

### 3.4. Analysis of the Secondary Structure and Stability of EgPI Using Circular Dichroism

Circular dichroism (CD) assays were conducted under varying pH and temperature conditions to analyse the secondary structure, and discern structural alterations in diverse environments. EgPI displayed a characteristic spectrum consistent with proteins featuring antiparallel β-sheets, aligning with the typical spectral pattern observed in Kunitz inhibitors [33]. The CONTIN algorithm suggests that EgPI is predominantly composed by β-sheets, random coils, and loops, with minimal α-helix content. This result is consistent with the information documented in the literature [23,31].

Dithiothreitol (DTT) induced noise and errors during spectral acquisition at elevated concentrations. Therefore, the concentration of DTT was held constant at ≤1 mmol·L^−1^. Under reducing conditions, EgPI exhibited a reduction in β-sheet content, and a marginal increase in α-helix, random structures, and loops. The changes in structural conformation reveal a shift from the original form, resulting in a loss of inhibitory properties. Two polypeptide chains emphasise this: the reactive sites only becomes active when the chains are close together.

Kunitz inhibitors possess high thermal stability [25]. Since a signature spectrum was determined under reducing conditions, we adopted this pattern to compare the secondary structure of EgPI across a range of temperatures. At high temperatures, the signal at ~200 nm was more positive than at lower temperatures, and at ~230 nm, the signal turned negative. At 70 °C, the α-helix content increased, and β-sheets decreased, a pattern observed at 80 °C and 90 °C. Mills et al. [34] described the reduction in β-sheets with the increase in α-helix content followed by the heat denaturation in soya globulin β-conglycinin. The heat denaturation increases surface hydrophobicity, decreasing the aggregate formation. As a result, there is an observed increase in the α-helix content [35].

The collected spectra of EgPI at different pH values exhibited only slight variations, implying the absence of discernible alterations in secondary structure within the pH range of 2 to 8. However, a significant change in the secondary structure was observed at pH 10, signifying minor alterations under alkaline conditions. Owing to its stability across different pH levels, EgPI has the potential for biotechnological application in pest control [23,36,37]. This is because of the presence of serine peptidases in the digestive systems of most coleopteran and lepidopteran pest insects, which function optimally in both acidic and alkaline conditions [38,39]. *Adenanthera pavonina* trypsin inhibitor (ApTI) retards the growth of *Anagasta kuehnella* (Lepidoptera) [29], and *Inga laurina* trypsin inhibitor (ILTI) exhibits insecticide activity against *Homalinotus coriaceus* [11] and retards the growth of *Spodoptera frugiperda* [40]. Employing transgenic plants that express PI genes could protect against pests and pathogens. The recombinant ILIT, produced through heterologous expression, exhibited inhibition activity comparable to that of the native ILIT [41]. This finding supports the potential biotechnological application of plant PIs because of their similar simple secondary structures and fewer post-translational modifications.

### 3.5. Antifungal Activities

Human pathogenic fungi, which represent only 0.1% of the described species, infect thousands of people yearly; the vast majority of these are mild infections. However, the incidence of hospital-acquired fungal infections has increased over time, leading to a rise in mortality rate, with candidiasis being the most common cause [15]. The frequency of certain types of nosocomial fungal infections is related to different degrees of immunosuppression. Immunosuppressive patients, including those with neutropenia, human immunodeficiency virus (HIV), chronic immunosuppression, transplants, burns, diabetes, and those undergoing therapy, are particularly at risk [42].

Studies have shown that compounds isolated from plants, such as PIs, can display antimicrobial activity [14]. Research on Kunitz inhibitors has revealed that their antifungal activity operates through multiple mechanisms. These include inhibiting proteases, inducing oxidative stress by interacting with mitochondria, blocking serine peptidases that prevent apoptosis (like metacaspases), and targeting nuclear mediators of apoptosis-like Nma111p [43].

EgPI exhibited notable antibiofilm activity against biofilm-forming strains of *C. albicans* and *C. tropicalis*. The antibiofilm activity of Kunitz inhibitors has been recently reported, where CITI at 2.1 μmol·L^−1^ showed a 34.30% reduction in biofilm formation and a 38.58% reduction in mature biofilm of *C. albicans* [13]. Besides CITI, other inhibitors have shown antibiofilm activity against various microorganisms. ILTI inhibited and prompted the eradication of mature *Staphylococcus epidermidis* biofilm at 25 and 150–200 μg·mL^−1^, respectively [44]. Against fungal yeasts, ILTI completely inhibited the growth of *C. tropicalis* and *C. buinensis* at 125 µg·mL^−1^ and 250 µg·mL^−1^, respectively [14]. *E. timbouva* trypsin inhibitor (EtTI) was tested against different planktonic yeasts, showing the highest effectiveness against *C. albicans*, moderate effectiveness against *C. buinensis*, and the most negligible effectiveness against *C. tropicalis* [15]. The effectiveness of EpTI was evaluated against biofilm at two different concentrations, specifically 1-fold and 10-fold MIC. The results revealed a 38% and 66% reduction for biofilm produced by *S. aureus*, and 40% and 74% for biofilm produced by *K. pneumoniae*, respectively. A reduction was only noticed with mature biofilm at ten times the minimum inhibitory concentration (MIC). This resulted in a 50% reduction in *S. aureus* and a 59% reduction in *K. pneumoniae*. In contrast with amphotericin B [45], EgPI displayed antibiofilm activity.

## 4. Materials and Methods

### 4.1. Materials

*E. gummiferum* seeds were purchased from Arbocenter Comércio de Sementes Ltda (São Paulo, Brazil). Bovine serum albumin (BSA), Nα-benzoyl-DL-arginine p-nitroanilide hydrochloride (BApNA), and succinyl-Ala—Ala—Pro—Phe-p-nitroanilide (SAAPFpNA) were bought from Sigma-Aldrich (São Paulo, Brazil). The electrophoresis reagents and rainbow high-range molecular weight marker were purchased from Cytiva (São Paulo, Brazil). All other chemicals and reagents used were of analytical grade.

### 4.2. Purification of E. gummiferum Peptidase Inhibitor

The seed coat of *E. gummiferum* was removed with a blade, and then the seeds were ground in an electric mill to prepare the seed powder. Protein extraction from the seed powder was conducted using 0.1 mol·L^−1^ sodium phosphate buffer (pH 7.6) containing 0.1 mol·L^−1^ of NaCl (1:10 *w*/*v*) overnight at 4 °C. The sample was centrifuged at 10,600× *g* for 30 min at 4 °C. The supernatant fraction was dialysed against distilled water for 24 h and then lyophilised to collect crude extract (CE), assayed for inhibitory activity against trypsin.

For gel filtration chromatography, 250 mg of CE was dissolved in 2 mL of 0.02 mol·L^−1^ ammonium bicarbonate buffer (AMBIC) and applied to a Sephadex G-100 column (3 × 80 cm) (Cytiva, São Paulo, Brazil) previously equilibrated with the same buffer. Fractions of 5 mL were collected at 18 mL·h^−1^. The fractions exhibiting inhibitory activity were dialysed, lyophilised, and named EgPI G-100.

For hydrophobic interaction chromatography, 250 mg of EgPI G-100 was dissolved in 5 mL of 0.1 mol·L^−1^ sodium phosphate buffer containing 0.4 mol·L^−1^ of ammonium sulphate (pH 7.2) and loaded onto a HiPrep 16/10 Phenyl HP column (Cytiva, São Paulo, Brazil), equilibrated with the same buffer at a flow rate of 5 mL·min^−1^. Inhibitory activity assays were conducted on the collected peaks, which were automatically collected using an Akta Pure 25 M system (Cytiva, São Paulo, Brazil). The peak exhibiting inhibitory activity was dialysed, lyophilised, and analysed using SDS-PAGE (Cytiva, São Paulo, Brazil) to confirm the molecular homogeneity of the sample.

### 4.3. Protein Quantification

The protein content was determined using the Bradford method [46], with bovine serum albumin (BSA) (Sigma-Aldrich, São Paulo, Brazil) at 1 mg·mL^−1^ as the standard.

### 4.4. Polyacrylamide Gel Electrophoresis

Sodium dodecyl sulfate–polyacrylamide gel electrophoresis (SDS-PAGE 12.5%) was performed as described by Laemmli [47]. The samples were mixed with the sample buffer, and the run was performed at a constant voltage of 100 V for 60 min. Protein staining was performed using Coomassie Brilliant Blue R-250.

### 4.5. Inhibitory Activity

The assays against trypsin were conducted according to Oliveira et al. [23]. An aliquot of 4 µL of trypsin (0.25 mg·mL^−1^) and 62 µL of 50 mmol Tris-HCl, pH 8.0, was mixed with 4 µL of different concentrations of EgPI, followed by incubation for 10 min at 30 °C. A volume of 200 µL of 1 mmol·L^−1^ BApNA was added, and the hydrolysis of the substrate was accompanied at 410 nm for 30 min, with readings taken at 5 min intervals on a Varioskan LUX microplate reader (Thermo Scientific, Sao Paulo, Brazil). One Trypsin Inhibitor Unit (TIU) was defined as the amount of inhibitor that decreased the absorbance by 0.01 units at 410 nm.

The assays of inhibitory activity against chymotrypsin were performed using an adapted procedure from Delmar et al. [48]. An aliquot of 4 µL of chymotrypsin (0.25 mg·mL^−1^) was mixed with 62 µL of 50 mmol·L^−1^ Tris-HCl, pH 8.0, and 4 µL of different concentrations of EgPI. This mixture was incubated for 10 min at 30 °C. Then, 20 µL of 1 mmol·L^−1^ SAAPFpNA was added, and the hydrolysis of the substrate was monitored at 410 nm for 7 min, with readings taken at 1 min intervals on a Varioskan LUX microplate reader (Thermo Scientific, São Paulo, Brazil).

### 4.6. Determination of Kinetic Parameters and Dissociation Constant (Ki)

To determine the kinetic parameters and dissociation constant (K*i*), various concentrations of EgPI (0.021, 0.0105, 0.00525, 0.002625, and 0.001312 mmol·L^−1^) and BApNA (0.5, 1.0, and 3.0 mmol·L^−1^) were used. The stoichiometry ratio between EgPI and trypsin was determined using the trypsin inhibitory activity assay. Both Dixon and Michaellis–Menten plots were employed to analyse the data (see Supplementary Information). Assays were performed at stoichiometric ratios of 0, 0.25, 0.5, and 1.0 between trypsin and EgPI. Each experiment was conducted three times independently, with each assay performed in triplicate.

### 4.7. Amino-Terminal Sequencing

The amino-terminal sequence of EgPI was determined in a Shimazu PPSQ-33B automated protein sequencer (Shimadzu, Kyoto, Japan) through Edman degradation. Phenylthiohydantoin (PTH) amino acids, the derivatives of amino acids liberated during sequence analysis, were detected at 269 nm on a Wakopak**^®^** Wakosil PTH-II (4.6 mm × 250 mm) column (Wako Fujifilm, Osaka, Japan). The alignment was searched using the NCBI-Blastp search system [49]. Multiple sequence alignment was performed using T-Coffee expresso (Center for Genomic Regulation, Barcelona, Spain) [50].

### 4.8. Circular Dichroism Spectral Analysis

EgPI was subjected to Far-UV CD spectral analysis using a JASCO J-1100 (JASCO, Tokyo, Japan). The secondary structure of EgPI (33 µmol·L^−1^) was initially determined in 10 mmol·L^−1^ phosphate buffer, pH 7.6, at 25 °C. The inhibitor was subjected to temperatures ranging from 20 to 90 °C to achieve a thermal denaturation curve. Each temperature was followed by a 10 min incubation period before collecting the spectrum.

To analyse the secondary structure conformation under reducing conditions, EgPI was incubated with 1 mmol·L^−1^ DTT. The secondary structure of EgPI was investigated under various pH conditions using different buffer solutions: 10 mmol·L^−1^ sodium citrate buffer at pH 2 and 4, 10 mmol·L^−1^ phosphate buffer at pH 6 and 8, and 10 mmol·L^−1^ sodium bicarbonate at pH 10. The scan spectra (190–250 nm) were collected in 1 mm quartz cuvettes with a scan rate of 50 nm·min^−1^, a 0.1 nm range, and a Data Integration Time (DIT) of 4 sec. Each spectrum collected resulted in 10 accumulations. The percent of the secondary structure was predicted using the Dichroweb server (Centre for Protein and Membrane Structure and Dynamics, Oxford, UK) through the CONTIN algorithm.

### 4.9. Microorganisms

Six American Type Culture Collection (ATCC) strains were used to analyse the antifungal activity of EgPI: *Candida albicans* ATCC 5314, *Candida albicans* ATCC 90028, *Candida glabrata* ATCC 90030, *Candida guillermondii* ATCC 6260, *Candida parapsilosis* ATCC 22019, and *Candida tropicalis* ATCC 750. All strains were stored at −80 °C until use. The yeasts were grown on Brain Heart Infusion (BHI) agar at 35 °C for 24 h. Following incubation, 1.5 × 10^6^ yeast cells were incubated in the suitable growth medium.

### 4.10. Antifungal Activity Assays

The minimum inhibitory concentration (MIC) and minimum fungicidal concentration (MFC) were defined according to the M27-A3 and M27-S3 Clinical and Laboratory Standards Institute (CLSI) protocol [51]. During the experiment, 1.5 million yeast cells were cultured in Sabouraud broth with EgPI concentrations ranging from 25 to 0.25 μmol·L^−1^. The assays were performed in 96-well microplates at 30 °C for 24 h, with amphotericin B as a positive control.

MIC was defined as the lowest concentration of EgPI capable of inhibiting visible microbial growth at the 96-well microplate. MFC was determined to have the lowest concentration of EgPI and did not allow visible microbial growth on solid agar after the incubation period.

### 4.11. Evaluation of Biofilm Properties of EgPI

The antibiofilm assays to inhibit and eradicate the yeast biofilm of *C. albicans* ATCC 90028 and *C. tropicalis* ATCC 750 were adapted from the methodology proposed by Filloux and Ramos [52]. A 20 μL aliquot of inoculum (1 × 10^8^ CFU^−1^mL^−1^), and 180 μL of BHI with 0.1% glucose were added to the wells of a 96-well microplate. Amphotericin B was used as a standard drug, and a sterile culture medium was included in microplates as a negative control of biofilm growth.

For the assay of the inhibition of biofilm formation, the microplate was incubated at 37 °C for 2 h to allow the pre-adhesion of cells. Following the period of biofilm incubation, the BHI medium was removed, the wells were washed with 0.9% sterile saline to remove planktonic cells, and the adhered biofilm was then treated with EgPI 11 μmol·L^−1^ and 5.5 μmol·L^−1^. A further period of incubation of 24 h at 37 °C was performed. The BHI medium was removed, the wells were washed with 0.9% sterile saline to remove planktonic cells, and the microplate was dried at room temperature for 2 h. Following drying, each well received 125 μL of crystal violet solution (0.1%), which was incubated at room temperature for 10 min. After the incubation, the supernatant of the crystal violet solution was removed, the wells were washed with 0.9% sterile saline, and 150 μL of 30% acetic acid was added to each well and incubated at room temperature for 10 min. Finally, the contents of the wells were homogenised, and 125 μL was transferred to a new flat-bottom plate with a lid.

To assay the effects of EgPI on mature biofilm, the microplate was incubated at 37° for 24 h to induce biofilm formation. After this incubation period, the BHI medium was removed, and the wells were washed with 0.9% sterile saline to remove planktonic cells. The adhered biofilm was then treated with EgPI 11 μmol·L^−1^ and 5.5 μmol·L^−1^. Another incubation period of 24 h at 37 °C was performed. Following this, the BHI medium was removed, the wells were washed with 0.9% sterile saline to remove planktonic cells, and the microplate was dried at room temperature for 2 h. Each well received 125 μL of crystal violet solution (0.1%), which was incubated at room temperature for 10 min. After the incubation, the supernatant of the crystal violet solution was removed, the wells were washed with 0.9% sterile saline, and 150 μL of 30% acetic acid was added to each well, and incubated at room temperature for 10 min. Finally, the contents of the wells were homogenised, and 125 μL was transferred to a new flat-bottom plate with a lid. The density of cells in suspension was determined at 550 nm on a Varioskan LUX microplate reader (Thermo Scientific, Sao Paulo, Brazil).

### 4.12. Analysis of Biofilm by Fluorescence Microscopy

To investigate the antibiofilm properties of EgPI using a second approach, biofilm was grown on a 6-well microplate, as described earlier. After washing, the biofilm was stained using SYTO-9 and propidium iodide (PI) from the LIVE/DEAD Cell Imaging Kit (Thermo Fisher Scientific, São Paulo, Brazil). SYTO-9 stains viable cells, while PI stains dead cells. After a 10 min incubation, images were captured using a fluorescence microscope (Leica DM2000 LED) equipped with a Leica DFC7000 T camera and LAS V4.12 software (Leica Microsystems, São Paulo, Brazil), using specific dye filters to distinguish between viable and dead cells.

### 4.13. Statistical Analysis

All statistical analyses were performed using GraphPad Prism 9.0 (Dotmatics, Boston, MA, USA). Data were analysed using a one-way ANOVA, with *p*-value ≤ 0.05 considered significant.

## 5. Conclusions

Fabaceae seeds are a rich source of PIs, particularly Kunitz and Bowman–Birk inhibitors. We outlined the purification method of EgPI, a Kunitz inhibitor that exhibits antibiofilm properties. Our research displayed that EgPI exhibits inhibitory solid effects on trypsin and displays a predominant secondary structure composed of β-sheets, characteristic of Kunitz inhibitors. The structural analysis revealed that EgPI maintains stability across a broad pH range, although its conformation changes under reducing conditions and elevated temperatures. The EgPI spectrum showed a decrease in β-sheet content and an increase in α-helix, random structures, and loops under reducing conditions. At higher temperatures, the β-sheet content decreased further, aligning with patterns observed in other heat-denatured proteins. The antimicrobial and antibiofilm activities of EgPI, evidenced by its effect on *C. albicans* and *C. tropicalis*, highlight its potential in biotechnological applications. When compared to other Kunitz inhibitors, such as CITI and ILTI, EgPI exhibits comparable or superior efficacy. These attributes underscore the multifaceted potential of EgPI, positioning it for diverse applications, including pest control, fungal management, and broader biotechnological research. The revealed bioactivity and stability of EgPI position it as a promising candidate for advanced development and application in these specific fields.

## Figures and Tables

**Table 1:** Purification of EgPI. The purification was performed starting with 200 mg of crude extract.

Steps	Total Protein (mg)	Total Activity (TIU) × 10^5^	Specific Activity (TIU·mg) × 10^4^	Yield (%)	Purification (Fold)
Crude extract (CE)	100.00	12.62	1.26	100.00	1.00
Sephadex G-100	21.42	10.27	4.80	81.38	3.80
Phenyl HP	5.41	7.13	13.17	56.46	10.44

**Table 2:** Amino-terminal sequence alignment of EgPI with Kunitz inhibitors showing the highest sequence identity: EcTI, *E. contortisiliquum* trypsin inhibitor; AcTI, *A. confusa* trypsin inhibitor; PjTI, *P. juliflora* trypsin inhibitor. Identical residues are highlighted in grey, and conserved substitutions are highlighted in dark grey. Similarity is represented as follows: (*) identical residues; (:) residues with high similarity; (.) residues with low similarity; and no marking for residues without similarity.

Inhibitors	Amino-Terminal Sequence	Identity (%)
EgPI	SELLDSDGDILDAGGAYYALPAVVS	
EcTI	KELLDSDGDILRNGGTYYILPALRG	68
AcTI	KELLDADGDILRNGGAYYILPALRG	64
PjTI	QELLDVDGEILRNGGSYYILPAFRG	60
Similarity	.**** **:** **:** ***. .	

**Table 3:** Determination of the secondary structure of EgPI under different conditions using circular dichroism.

Conditions of Incubation	Secondary Structure Content (%)			
	α-Helix	β-Sheets	Random Coils	Loops
EgPI	2.9	21.6	45.7	29.8
EgPI + DTT (1 mmol·L^−1^, 30 °C, 30 min)	7.6	8.3	49.5	34.6
EgPI at 20 °C	6.6	38.3	32.9	22.2
EgPI at 30 °C	6.6	38.3	32.9	22.2
EgPI at 40 °C	9.9	14.4	40.7	35.0
EgPI at 50 °C	9.0	16.4	40.9	33.7
EgPI at 60 °C	9.2	15.1	42.1	33.5
EgPI at 70 °C	25.6	8.4	33.7	32.3
EgPI at 80 °C	36.1	8.0	24.6	31.2
EgPI at 90 °C	38.0	5.4	31.9	24.7
EgPI at pH 2.0	5.4	13.3	46.2	35.0
EgPI at pH 4.0	10.5	10.9	39.7	39.0
EgPI at pH 6.0	8.6	13.5	44.1	33.8
EgPI at pH 8.0	5.2	17.1	44.6	33.2
EgPI at pH 10.0	7.6	9.7	48.2	34.5

**Table 4:** Antimicrobial properties of EgPI against pathogenic yeasts. MIC, minimum inhibitory concentration; MFC, minimum fungicidal concentration; MBIC, minimum biofilm inhibitory concentration; MBEC, minimum biofilm eradication concentration. (----) Represents that no antibiofilm assays were performed.

Strains	Planktonic		Biofilm	
	MIC (µmol·L^−1^)	MFC (µmol·L^−1^)	MBIC (µmol·L^−1^)	MBEC (µmol·L^−1^)
C. albicans ATCC 5314	>25	>25	1.125	5.5
C. albicans ATCC 90028	>25	>25	----	----
C. parapsilosis ATCC 22019	>25	>25	----	----
C. guillermonds ATCC 6260	>25	>25	----	----
C. tropicalis ATCC 750	>25	>25	1.125	>11
C. glabrata ATCC 90030	>25	>25	----	----

## Data Availability

The data presented in this study are available in the article and Supplementary Materials.

## Supplementary Materials

The following supporting information can be downloaded at: https://zenodo.org/doi/10.5281/zenodo.13207925 (accessed on: 4 August 2024), File S1: Michaelis Menten and Dixon plot of EgPI.
